# Nanohelices from planar polymer self-assembled in carbon nanotubes

**DOI:** 10.1038/srep30310

**Published:** 2016-07-21

**Authors:** Hongjin Fu, Shuqiong Xu, Yunfang Li

**Affiliations:** 1 College of Mechanical Engineering, Linyi University, Linyi, Shandong 276005, People’s Republic of China; 2 Key Laboratory for Liquid-Solid Structural Evolution and Processing of Materials, Ministry of Education, Shandong University, Jinan 250061, People’s Republic of China

## Abstract

The polymer possessing with planar structure can be activated and guided to encapsulate the inner space of SWNT and form a helix through van der Waals interaction and the π-π stacking effect between the polymer and the inner surface of SWNT. The SWNT size, the nanostructure and flexibility of polymer chain are all determine the final structures. The basic interaction between the polymer and the nanotubes is investigated, and the condition and mechanism of the helix-forming are explained particularly. Hybrid polymers improve the ability of the helix formation. This study provides scientific basis for fabricating helical polymers encapsulated in SWNTs and eventually on their applications in various areas.

Increasingly, the noncovalent “wrapping” and “encapsulating” of polymer chains on carbon nanotubes (SWNTs) are interesting phenomena, which have been widely studied in theoretical and experimental researches^1^,^2^,^3^,^4^. The polymer adhered to SWNTs can aid the solubility and dispersion of intact SWNTs^5^,^6^,^7^, enhance the mechanical strength, electrical conduction, and optical nonlinearity of the polymer matrix^8^,^9^ and functionalize the SWNTs^10^,^11^. Moreover, the noncovalent polymer stacking (CH-π or π–π) on SWNT surface controlled by the balance of attractive/repulsive forces within and between molecules can drive self-assembly mechanisms^12^,^13^. Self-assembly can result in truly unique and highly ordered structures that such materials may not only be designed to be highly dynamic, displaying adaptive and self-healing properties, but also help gain an understanding of the rules that govern molecular assembly processes^14^.

The existence of helical folding in biopolymers like proteins and nucleic acids is very important, which direct the sophisticated functions in living systems. Inspired by biological helices, the polymers are expected to be designed with controlled helicity functions^15^,^16^,^17^,^18^ to design elongated supramolecular architectures. The new and reliable method for unambiguously constructing and determining single- and double-stranded helical polymers are important and urgent challenges in this area. The single- and double-helical structure confined within a SWNT is of significant importance, which may provide a new system that mimics the unique adaptive, dynamic and complex molecular models found in nature^19^,^20^. It is the new technology and ability to design and enforce a one-dimensional helical morphology within the hollow space of SWNT^21^,^22^,^23^, which will result in diverse functional polymer-SWNT complexes for a wide range of applications in electronics and biomedicine.

The present study discovers that the polymers, which possessing planar configuration, can self-assemble within SWNTs to form single- and double-helix. The SWNT surface provides adsorption stress to bend polymer chains such that they attach onto the inner surface of SWNT and roll up to self-assemble. The SWNT size, the flexibility and nanostructures of polymer chain are all determine the outcome of the nanostructures. To fully understand the potential of such functional systems, the basic interaction between the polymer and the nanotubes is investigated, and the condition and mechanism of the helix-forming are also particularly explained. Of significant importance is that this study provides scientific basis for designing and fabricating helical polymers encapsulated in SWNT and eventually on their applications in various fields.

## Results

**Figure 1:**
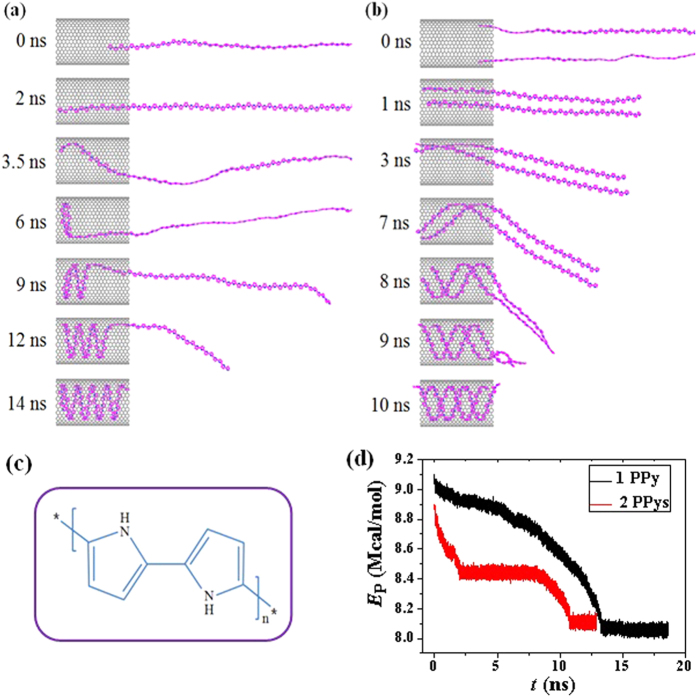
(**a**) The helical encapsulation of a PPY into the (20, 20) SWNT; (**b**) The double-helix is formed when two polymers inserting into the (20, 20) SWNT; (**c**) The repeat unit for the rigid backbone of PPY; (**d**) Total potential energy (*E*p) of these two PPY-SWNT systems as a function of time.

The present work aims to reveal the helix-forming process, especially the condition and mechanism of the polymer when encapsulating into the SWNT at an atomic level. Polypyrrole (PPY) is a type of organic polymer, which is widely used in electronic devices and chemical sensors^24^. The Nobel Prize in Chemistry was awarded in 2000 for work on conductive polymers including PPY^25^. Our simulations show that the PPY with strict planar construction can easily form single- and double-helix inside the SWNT. Direct simulations in Fig. 1 provide the representative snapshots of one and two PPY chains helically inserting into the SWNT (20, 20) (See the video 1 in the Supporting Information for the detailed inserting processes). The inset (a) is the repeat unit for the rigid backbone of PPY. The length of the SWNT is 49.19 Å and the PPY is built in a head-to-tail configuration with the length of 300.61 Å. Because of natural curl characteristics, the single PPY chain twists or bents in a free space showing one-dimensional wavy fluctuation like a worm.

If one end of the PPY is captured by the inner space of SWNT and the deformation force from the PPY itself is overcome, the polymer adheres onto the tube wall quickly owing to the vdW coupling. As the simulation progresses, interactions from the so-called vdW potential well gradually pull the polymer chains inside the SWNT in a straight line (t = 0~2 ns), in which the PPY are held tightly against the sidewall of the tube and the fluctuations vanish owing to the interaction between them. After reaches to the end of the tube, The chain are then pushed further inwards by the parts still located outside the tube, which continue to enter the SWNT in a helical fashion. While the PPY head has formed a helical configuration with large pitch instead of overflow the tube. The PPY forms a coil when the time is up to 7 ns. Then the PPY heads are pushed to move forward circumferentially because of the curvature of the tube wall. Eventually, a perfect single-helix, with remarkably constant gap of 3.5 Å between neighboring spirals, fills up the SWNTs at 10 ns. The self-assembled PPY-SWNT system achieves dynamic equilibrium through spontaneous insertion. The handedness of the PPY helix is determined by the initial deflection of the captured end, which can be right- or left-handed with equal probability.

Double-helix can also be formed when two PPY chains encapsulating into the (20, 20) SWNT, the evolution process is illustrated in Fig. 1(b) (see Video 2 in Supporting Information for detailed encapsulation process). Two PPY chains are separated for about 2 nm at one end of the tube. As the simulation starts, two PPY chains contact each other rapidly through π-π interactions between them. They also fill the tube in a straight way due to the effect of vdW potential well and, the head of the polymer chains reach the other end of the SWNT after 1 ns. Then the polymer chains begin to bend into a helical shape within the confined space at 7 ns. Eventually, a perfect double-helix with equal periodicity forms inside the SWNT at 10 ns. Two polymer chains always contact each other during the filling process, suggesting that the π-π stacking effect between the two chains should be stronger than that in the PPY-SWNT system. It is should noting that the double-stranded helical polymer is similar to the DNA. The similarity could be a clue to the potential applications of this double-helix structure and one of the most heralded inorganic nanomaterials.

**Figure 2:**
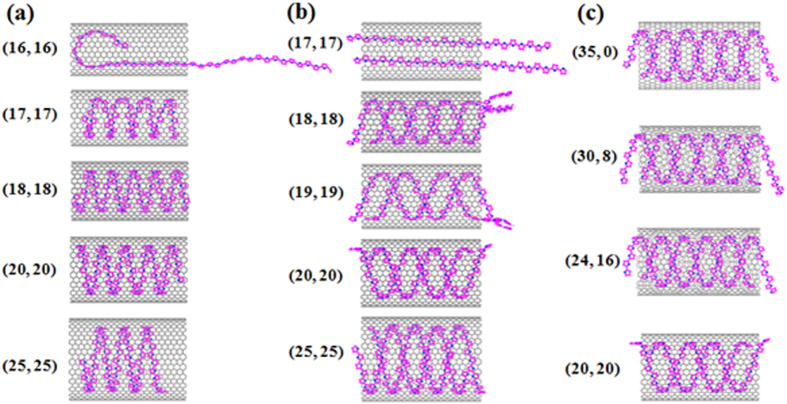


In addition, the dependence of the helix formation on diameter and chirality of SWNT has been further studied. It has known that the chirality of SWNTs have a significant effect on their properties, which can change from metallic when they adopt an armchair chirality to semiconducting or semimetallic when they are zigzag or chiral^26^. Figure 2(a,b) show that a helical structure is obtained for one and two polymer chains in the (16, 16) and (17, 17) armchair SWNTs, respectively. Whereas in SWNTs (15, 15) and (16, 16) with smaller diameters the polymer keeps curving or straight pattern for one and two PPYs. If the diameter of the SWNT is smaller enough, the polymer cannot be encapsulated in the nanotube. As the diameter increases, the polymer chains can produce a perfect single and double helix inside the SWNT and polymer helix with a constant pitch to reduce the curvature of chains and keep the whole system stable. To investigate the effect of chirality on the system, four types of SWNTs including (35, 0), (30, 8), (24, 16), and (20, 20) are chosen, which have different chiral angles θ ranging from 0 to 30° and similar diameters about 22.7 Ǻ. The chiral angle θ and corresponding diameter of the SWNTs with (n, m) indices can be determined by using the rolling GN model^27^. The total number of atoms, diameter, and length of each chiral nanotube are nearly the same. Figure 2(c) shows the final helical nanostructures of the polymer molecules in SWNTs after the MD simulations. There is a slight effect of the chiral angles θ on the pitch of the helical nanostructures and the distances of the adjacent gaps was kept constant within 9.4 to 9.6 Ǻ.

**Figure 3:**
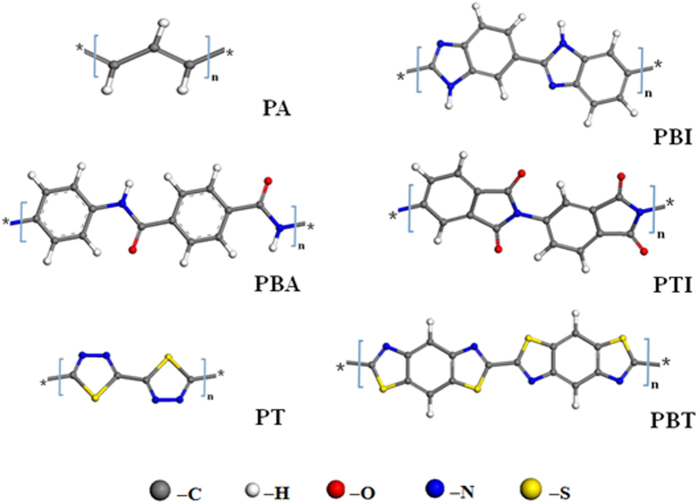
The repeat unit of the planar polymers used in this study. Refer to Table 1 for the full information of the polymers. Compare to flexible PE, PA, PPY and PBI have moderate flexibility backbones, PBA and PTI have more flexibility backbones, PT and PBT are considered nearly as rigid polymers due to the C-S bond of the backbone.

**Figure 4:**
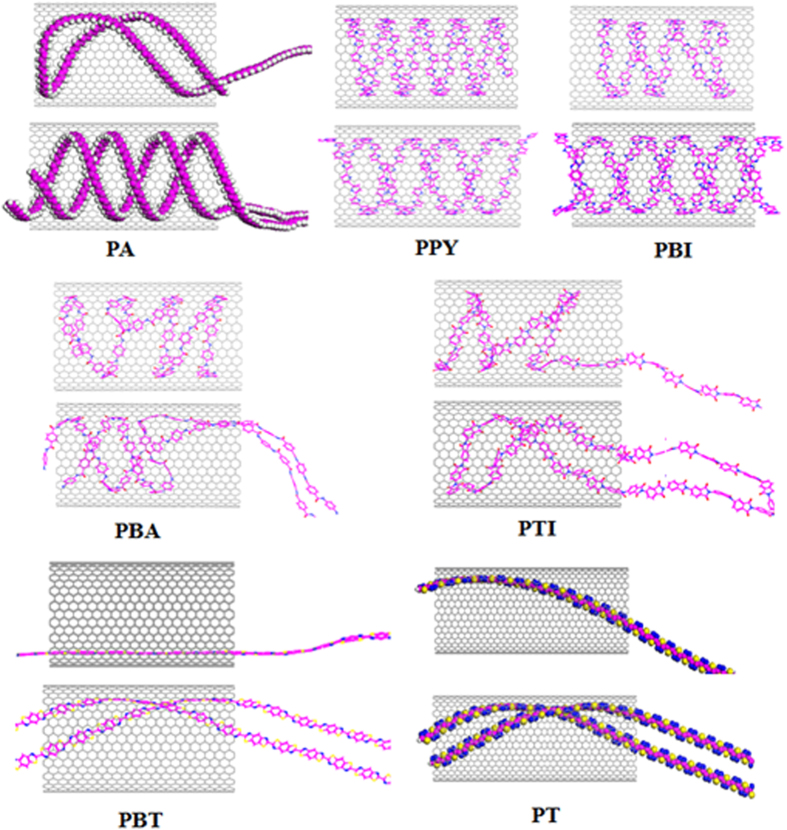


The interaction of SWNT and some non-planar and planar polymers are simulated. The repeat unit of poly(thiodiazole) (PT), Poly(p_benzamide) (PBA), Poly(p_thalimide) (PTI), Poly(benz_imidazoles) (PBI), Polyacetylene (PA), Poly(benzothiazole) (PBT) with planar structure are shown in Fig. 3. Table 1 gives the full information of these polymers used in this study. As expected, only these polymers with planar configuration can encapsulate the SWNT and show some helical structure more or less when the system saturates. Some correlations of helical bending to their corresponding polymeric species can be seen from the molecular conformations at 20 ns that shown in Fig. 4.

**Figure 5:**
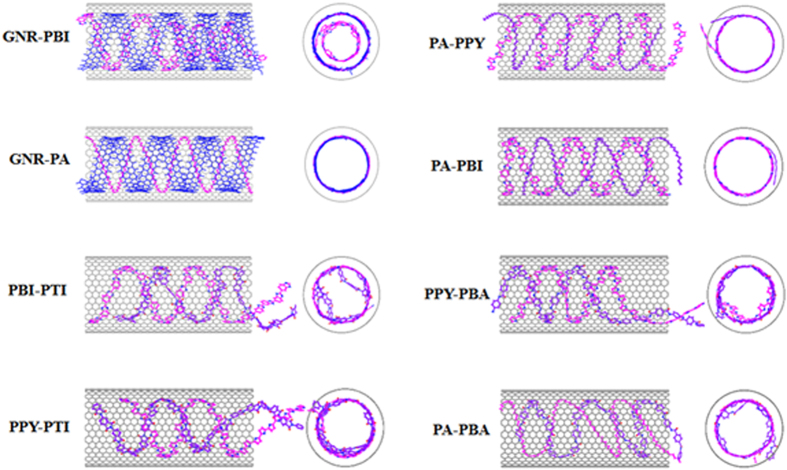


Considering that nanodevices may be need more species of polymer, it endeavors to rationally design hybrid helix in nanotubes, which will result in responsive, multiplexed and photostable polymer probes. Figure 5 shows the formed configuration of hybrid helix in SWNTs. Several recent reports has demonstrated that graphene nanoribbon (GNR) is able to encapsulate into the SWNT and form a perfect helical structure spontaneously as a result of the π–π stacking between GNR and SWNT^28^,^29^,^30^,^31^. This peculiar phenomenon prompts us to design the GNR-polymer hybrid helix. The GNR utilized was 7.38 Å in width and the SWNT (20, 20) was 59.03 in length. GNR with polymers PA and PBI can form perfect double-helix. If the PBI is long enough, its end will insert the inner space of GNR helix to form a hybrid double-walled helix. In the helix of GNR and PA, the PA chain has more intense vibration than that of GNR. It is notable that two different polymers are easier to form hybrid helix showing that hybrid improves the ability of the helix-forming.

## Discussion

**Figure 6:**
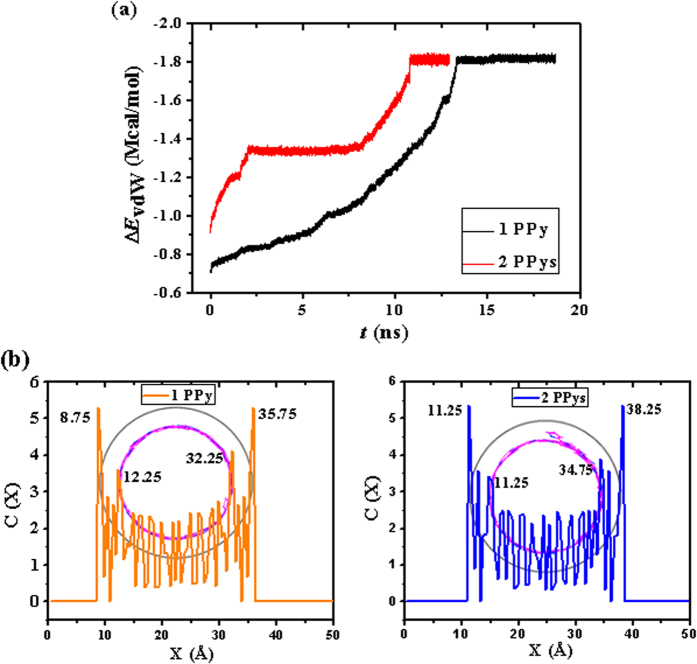
(**a**) The evolution of vdW interaction energy (∆*E*_vdW_) between the PPYs and SWNTs as a function of time. (**b**) Concentration distribution profiles of the core-shell composite structure formed from the PPYs and SWNTs in the X-direction.

It is essential to answer why the planar polymer can encapsulate into the SWNT spontaneously and stick on the tube wall in a spiral manner. Theoretically, the short polymer exhibits a worm-like chain conformation sustained by its intrinsic bending strain energy, though the polymer will tend to agglomerate driven by thermal fluctuation and lose orientational order when it is very long. The deformation and helix-forming are attributed to the competition between the vdW interaction energy and the bending strain energy of the polymer, which always tends to keep the intrinsic chain form and provide an energy barrier to structure transition. When the SWNT approaches, the attractive vdW force can help the polymer overpass the energy barrier to deform. During the self-assembly, the potential energy of the system transits into vdW attraction between polymer and SWNT. As illustrated in Fig. 6(a), the negative Δ*E*_vdW_, indicating an attractive force, has increased dramatically and finally reaches the highest energy states, which are changed nearly synchronous with that of *E*_P_ during the whole helix-forming process. In this course, the vdW is partially converted into the internal energy, which drives the geometry deformation of polymer, and partially transforms to kinetic energy, which sustains the structure transition. Therefore, the vdW interaction between the polymer and the SWNT plays a dominant role in helix-forming.

**Figure 7:**
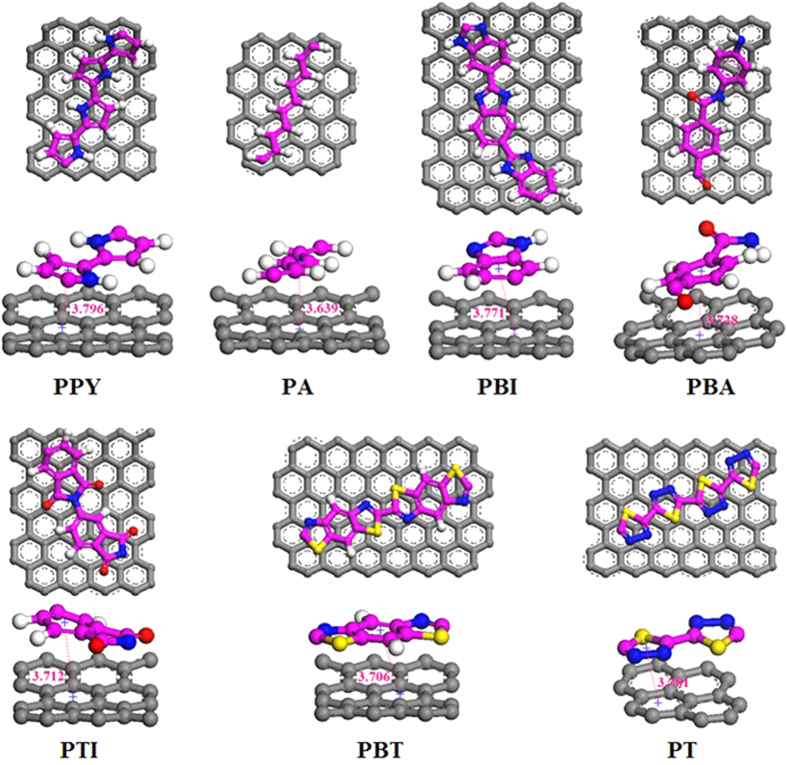


The offset π-π stacking interaction^32^ between the polymer and the SWNT also plays a significant role in the helical self-assembly. It can be validated from the concentration distribution profiles of the composite structures in the X directions (Fig. 6(b)) that the separation between polymer plane and SWNT is exactly close to 3.5 Å, which in accordance with the parallel stacking distance of the offset π-π stacking interactions. The partial enlarged details of the helical polymers inside SWNTs in Fig. 7 also demonstrate that the ring planes of polymers is offset from SWNTs’ and their centroid-centroid distances are all between 3.6 and 3.8 Å, which is lower in energy than other face-face and T-shaped stacking arrangements according to the typical aromatic-aromatic offset π-π stacking^33^. It speculates that the offset π-π stacking interactions can provide two key elements for the formation of a helix, one is an energetic contribution that stems from the stacking itself, and such a contribution can thermodynamically drive the helix-forming process; the other is specific directionality and orientation provided by the specific pattern of stacking^34^ that determines the parallel separations of polymer plane and SWNT. Thus, the best way to keep the displaced stacking and the most contact area of polymers and SWNT is that the arrangement of the polymer follows a helical mode and paralleled arrangement when increasingly longer polymer is trapped inside the tube. Moreover, the displacement of the carbon rings favors to minimize the repulsive π-π interaction and maximize the attractive π-σ interaction. Therefore, in the polymer-SWNT systems, parallel displaced offset π-π stacking should be the major organization of π-π interaction displacement and plays a dominant role in helix-formation. The driving forces for forming these configurations are most likely a combination of vdW force and π-π staking^28^,^35^. Firstly, the vdW interaction draws and traps the polymers inside the tube; then the offset π-π staking interaction guides the chains to self-assemble and display a helical configuration.

The most important factor is that the polymer must possess a planar structure to guide displaced stacking in the system. It is because the three-dimensional hydrogen bond and side-groups enlarge the distance between polymer and SWNT, which will destroy the displaced stacking and prefer to coil rather than form helix^36^, just as shown in Fig. 4. Moreover, the moderate flexibility of the polymer chains also contributes to helical formation ability of the polymer during encapsulation. PA has a relative stiff backbone, which make a PA chain fill the entire diameter of the SWNT and fold up into loops rather than helix. While two PAs restraining each other to enhance the flexibility, which combine with the noncovalent π-π stacking between PA and SWNT wall allowing them to form a perfect double helix. When the head of a single PA retraces and meet the other part of the PA, a helical pattern also presents (see Fig. 4). This π-π stacking is that the C-C double bonds of the PA interacts with the π-electrons of six-membered ring of SWNT. Though these π-π interactions of PA and SWNT is significantly weaker than π-π stacking between rings, C-C double bonds are very abundant and this π-π stacking can provide the crucial driving force in the helix-forming process of PA in SWNT.

While the too flexible polymer chains, such as poly (ethylene) (PE)^23^, prefer to coil up and agglomerate with intrachain interactions rather than interact with the SWNT. Because the T-shaped CH-π stacking between PE and SWNT is weaker than PE intrachain force, and can not drive PE to adhere onto SWNT wall and form helix. PBI and PPY possess moderate flexibility to form single- and double-helix, which retain their planar structure in the whole encapsulation. Aromatic moieties along the backbone of PBI and PPY prefer to optimize and enhance the π-π interactions and dictate the adsorbed helix conformation^37^. The nitrogenous rings of PPY and PBI strength the π-π stacking with six-membered rings in the SWNT and the introduction of nitrogen to the rings increases the tendency to stack, as shown in Fig. 7. Moreover, recent studies have suggested that a combination of the backbone stiffness and aromaticity (and thus π-π interactions with the SWNTs) can stabilize polymer adsorption to the SWNT surface and can also lead to enhanced functionalization^38^,^39^,^40^.

The inserted PBA and PTI chains are actively interacting with the SWNT wall and take a helical pattern. The ability of forming helix is lower than above three polymers. The reason is that the atoms N that join molecules make adjacent molecular structures relatively rotate. The rotation causes the faces of the neighboring molecules almost perpendicular. Though aromatic rings of polymer strengthen the π-π interactions with SWNT, the relatively rotation increases the flexibility of the chain backbones and enlarges the interaction distance between polymers and SWNT wall, which hinder the formation of helix. For PBT and PT, their stiff backbones latch onto the SWNT surface owing to the presence π-π interaction of five- and six-membered rings with six-membered rings in SWNT, has a greater tendency to bend in a helical pattern. However, C-S bonds reinforce the stiffness of the backbones, which make PT and PBT only have a helical tendency in the SWNT length and cannot bend anymore to form helix. In comparison, poly(styrene) (PS) and poly(ethylene terephthalate) (PET), though the aromatic rings make them latch onto the SWNT dominated by π-π interactions, the contained three dimensional C-H bonds weaken the interaction of the whole chain with tube wall and make the polymer coil up or form S-shaped arrangement along one side of the SWNT instead of helical conformation^23^.

Therefore, three factors should be considered to promote and enhance the formation of a perfect helical structure. (1) The polymer should possess a planar configuration, which will guide and strengthen the displaced stacking in the system; (2) the moderate flexibility of the polymer chains, which cause the chain to bend and form helix; (3) strong interactions between polymer and SWNT, which will act as the driving force for self-assembly.

This study provides a direct observation that the polymer with planar structure can be activated and guided to encapsulating the inner space of SWNT and form a new helix on a molecular scale. The vdW attraction drive the polymer to fill the SWNT and the π-π staking interactions between polymer and SWNT wall guide the chains to self-assemble to display a helical configuration and cause polymer and SWNT wall with the spacing of 3.5 Å. The diameter of the tube that PPY chains can form single- and double-strand helix should be larger than that of SWNT (17, 17) and (18, 18), respectively. But the SWNT chirality has a negligible influence on the helical-encapsulation. Hybrid double-helix has also been designed and hybrid improves the ability of the helix-forming. To obtain a perfect helix, the polymer should possess a planar configuration, because the three-dimensional hydrogen bond and side-groups enlarge the distance between polymer and SWNT, which will destroy the displaced stacking and prefer to coil rather than forming helix. Furthermore, the polymer should have moderate flexibility and strong interactions with SWNT. Of significant interest is that the proposed discoveries provide the theoretical basis for designing and fabricating single- and double-helix nanostructured polymers inside SWNTs via self-assembly and eventually on their applications in various areas.

## Methods

All calculations are performed using molecular dynamics (MD) simulation, which is an effective tool for studying material behavior at the nanometer scale and providing detailed information at the atomic level. The force-field of condensed-phase optimized molecular potentials for atomistic simulation studies (COMPASS)^41^ was applied to model the atomic interaction. The COMPASS is an *ab initio* force-field that is parameterized and validated using condensed-phase properties in addition to various *ab initio* and empirical data. It aims to achieve high accuracy in prediction of the properties of very complex mixtures^42^,^43^, and it has been proven to be applicable in describing the properties of graphene and SWNTs^44^,^45^ and the interaction of polymer and SWNT^46^,^47^. The Andersen method in the thermostat was applied to control the temperature and generate the correct statistical ensemble. The thermodynamic temperature was kept constant by allowing the simulated system to exchange energy with a “heating bath”. The initial velocities of all atoms followed a temperature-dependent Maxwell-Boltzmann distribution and the Verlet algorithm was adopted to integrate the motion of equations of the whole system.

In this study, all the MD simulations were performed in vacuum at 298 K and different SWNTs and polymer chains were studied. Each of the system was simulated long enough to achieve an equilibrium state. The SWNTs were frozen as rigid structures to simplify the computational analysis of the run. Initially, polymers were placed at the entrances of the SWNTs along the axial direction and overlapped 15 Å with the SWNTs in order to overcome the deformation force from the polymers themselves and insure the distances between them are in the range of the cutoff distance of van der Waals (vdW) interaction. Then the models were put into a canonical ensemble molecular dynamics (NVT-MD) simulation. The time step was set to be 1.0 fs. Data were collected every 5.0 ps to record the full-precision trajectory and the results were further analyzed.

## Additional Information

**How to cite this article**: Fu, H. *et al*. Nanohelices from planar polymer self-assembled in carbon nanotubes. *Sci. Rep.* **6**, 30310; doi: 10.1038/srep30310 (2016).

## Supplementary Material

Supplementary Video 1

Supplementary Video 2

Supplementary Information

## Figures and Tables

**Table 1:** Full information of polymers used in this study.

**Type of polymer molecules**	**Full name**	**Length (Å)**	**Type of atoms and atoms number**	**Molecular weight**
PPY	poly(pyrrole)	174.797	C_200_H_152_N_50_	3252
PA	Poly(acetylene)	234.960	C_200_H_202_	2602
PBI	Poly(benz_imidazoles)	166.106	C_196_N_56_H_114_	3250
PBA	Poly(p_benzamide)	187.821	C_196_N_28_O_28_H_142_	3334
PTI	Poly(p_thalimide)	154.017	C_200_N_25_O_50_H_77_	3627
PBT	Poly(benzothiazole)	196.010	C_200_N_50_S_50_H_52_	4752
PT	poly(thiodiazole)	193.624	C_100_N_100_S_50_H_2_	4202
PE	Poly(ethylene)	251.040	C_200_H_402_	2802

All the polymer molecules contain about 200 C atoms, and the length is the approximate end-to-end length. Considering the length of the polymer chains, PT is selected containing 100 C atoms.
