# Supplementary material for: Nanohelices from planar polymer self-assembled in carbon nanotubes
Source: Sci Rep. 2016 Jul 21;6:30310. doi: 10.1038/srep30310 (PMC4954971; doi:10.1038/srep30310)
Supplement: Supplementary Information [file srep30310-s3.doc]

**Nanohelices from planar polymer self-assembled in carbon nanotubes**

**Hongjin Fu^1^, Shuqiong Xu^1^ & Yunfang Li^1,2^**

**Additional Information**

Supplementary information accompanies this paper at <http://www.nature.com/srep>

**Video 1.** A PPY chain encapsulating into a SWNT (20, 20) to form a single-helix.

**Video 2.** Two PPYs inserting into a SWNT (20, 20) to form a double-helix.
